# Role of Stenting for Intracranial Atherosclerosis in the Post-SAMMPRIS Era

**DOI:** 10.1155/2013/304320

**Published:** 2013-11-20

**Authors:** Dale Ding, Robert M. Starke, R. Webster Crowley, Kenneth C. Liu

**Affiliations:** ^1^Department of Neurosurgery, University of Virginia, Charlottesville, VA 22908, USA; ^2^Neurological Surgery and Radiology, Division of Cerebrovascular and Skull Base Surgery, Department of Neurosurgery, University of Virginia Health System, P.O. Box 800212, Charlottesville, VA 22908, USA

## Abstract

*Introduction*. The initial promise of endovascular stenting for the treatment of intracranial atherosclerotic disease (ICAD) has been tempered by the results of the SAMMPRIS trial which demonstrated better outcomes with medical management compared to stenting for symptomatic ICAD. We review post-SAMMPRIS ICAD stenting outcomes. *Methods*. A comprehensive literature search was performed using PubMed to identify all ICAD stenting series published after the SAMMPRIS in September 2011. The type and design of the stent, number of patients and lesions, inclusion criteria, and clinical and angiographic outcomes were noted. *Results*. From October 2011 to August 2013, 19 ICAD stenting series were identified describing the interventional outcomes for 2,196 patients with 2,314 lesions. Of the 38 different stents used, 87% were balloon-expandable stents (BESs) and 13% were self-expanding stents. The median minimum stenosis was 50%. The median rates of technical success rate, postprocedural ischemic events, and symptomatic in-stent restenosis (ISR) were 98% (range 87–100%), 9.4% (range 0–25%), and 2.7% (range 0–11.1%), respectively. The median follow-up durations were one to 67 months. *Conclusions*. The management of severe ICAD remains controversial. Future trials are needed to define the optimal patient, lesion, and stent characteristics which will portend the best outcomes with intervention.

## 1. Introduction

Intracranial atherosclerotic disease (ICAD) accounts for approximately 10% of ischemic stroke in Western society and approximately 33–50% of stroke in Asia [1, 2]. Black and Hispanic ethnicities, diabetes mellitus, and metabolic syndrome predispose patients to developing ICAD [3]. The natural history of ICAD is significantly different for asymptomatic compared to symptomatic patients. The Warfarin-Aspirin Symptomatic Intracranial Disease (WASID) trial was a prospective, randomized study which demonstrated aspirin to have equivalent efficacy but superior safety to warfarin for the treatment of symptomatic ICAD [4]. Despite treatment with aspirin, the rate of ischemic stroke was 15% and 20% at one- and two-year followup, respectively. Coexisting asymptomatic ICAD lesions of 50–99% stenosis were detected in 27% of WASID study patients on magnetic resonance angiography [5]. The rate of ischemic stroke in the territory of the stenotic artery for symptomatic compared to asymptomatic patients was 12% versus 3.5% at one year, respectively [4, 5].

In order to improve the poor outcomes associated with symptomatic ICAD, endovascular revascularization of affected intracranial arteries utilizing stents has become popularized over the past decade [6]. Initial results were promising with high rates of technical success, excellent angiographic outcomes, acceptable complication rates, and noted reductions in posttreatment ischemic events compared to the natural history [7–9]. In 2011, the Stenting versus Aggressive Medical Management for Preventing Recurrent Stroke in Intracranial Stenosis (SAMMPRIS) trial was published and demonstrated superiority of medical therapy to endovascular stent intervention [10]. As a result, the validity of stenting for ICAD has become a subject of significant debate [11, 12]. Previous reviews describing ICAD stenting outcomes have primarily described studies published prior to SAMMPRIS [13–15]. It is unknown whether these interventional outcomes have changed following the publication of SAMMPRIS due to alterations in referral patterns from primary care physicians and neurologists, stricter patient selectivity from neurointerventionalists, and changes in stent preference including newer generation intracranial stents and bare metal and drug-eluting coronary stents. We review the stenting outcomes for ICAD in the post-SAMMPRIS era. 

## 2. Methods

Utilizing PubMed, we performed a comprehensive literature search for all endovascular stenting series for the treatment of ICAD following the publication of SAMMPRIS in September 2011 [10]. The terms “intracranial atherosclerosis,” “stent,” “stroke” and “endovascular procedures” were used to search for relevant publications. Single case reports and small case series comprising less than 10 patients were excluded. Case series including patients who received angioplasty alone without stent placement were also excluded [16]. The stent name, stent design, stent type, number of patients, number of ICAD lesions, minimum degree of arterial stenosis to be considered for stenting, technical success rate, duration of clinical followup, rate of postprocedural stroke (including TIAs) in the ipsilateral vascular territory and in all territories, and the rate of symptomatic in-stent restenosis (ISR) were noted when available. The stent design was classified as balloon-expandable stent (BES) or self-expanding stent (SES), and the stent type was classified as coronary, intracranial, or biliary.

## 3. Results

### 3.1. Stenting Outcomes for Intracranial Atherosclerosis in the Post-SAMMPRIS Era

From October 2011 to August 2013, we identified 19 single-center and multicenter case series.  Table 1 summarizes the ICAD stenting series published after SAMMPRIS [17–34]. The median number of patients per series was 60.5 (range 11–637), and the median number of treated lesions was 62 (range 11–670). A total of 2,196 patients with 2,314 ICAD lesions were treated with endovascular stenting. The average number of lesions treated per patient was 1.05. The vast majority of patients were treated for symptomatic ICAD although some studies also treated a small minority of patients with asymptomatic ICAD. The minimum stenosis of the diseased artery which warranted intervention was 50% in 11 series, 70% in six series, and not reported in one series (median 50%).

Technical success, typically defined as greater than 50% revascularization of the diseased artery, was very high in most series, with a median rate of 98% (range 87–100%). Over a median or mean follow-up period which ranged from one to 67 months, the median rate of ipsilateral ischemic events, including all TIAs and strokes, was 5.4% (range 0–13.7%), and the median rate of ischemia in any territory was 9.4% (range 0–25%). In-stent restenosis (ISR), typically defined as at least 50% recurrent stenosis of the stented arterial segment at follow-up angiographic evaluation, was classified as asymptomatic or symptomatic. Symptomatic ISR occurred at a median rate of 2.7% (range 0–11.1%). Of the 19 series, 11 utilized a single stent (57.9%) and eight utilized multiple stents (42.1%).  Table 2 lists the stents and their manufacturers. A total of 38 different stents produced by 14 manufacturers were used in the reviewed series. The stent designs included 33 BESs (86.8%) and five SESs (13.2%). The stent types were coronary in 27 (71.1%), intracranial in nine (23.7%), and biliary in two (5.3%). All coronary stents were BESs and six of the coronary BESs were drug-eluting (paclitaxel *N* = 3 and sirolimus, zotarolimus, and dexamethasone each *N* = 1).

### 3.2. Self-Expanding Intracranial Stents

The Wingspan stent (Boston Scientific, Natick, MA, USA) is a self-expanding nitinol stent which was approved by the Food and Drug Administration (FDA) in 2005 under a humanitarian device exemption (HDE) for patients with symptomatic, severe ICAD of at least 50% who have failed medical management with antiplatelet therapy [7]. The Wingspan stent system is used in conjunction with the Gateway percutaneous transluminal angioplasty (PTA) balloon (Boston Scientific) which is used to dilate the diseased segment of the artery prior to stent deployment. The Gateway PTA balloon and Wingspan stent system were used exclusively in the SAMMPRIS trial, and it was this specific SES which failed to demonstrate superiority over medical management [10]. Nonetheless, 12 of the 19 ICAD stenting studies we reviewed used the Wingspan. Seven studies used the Wingspan stent exclusively comprising 377 patients with 402 lesions [17, 19, 20, 25, 27, 30, 32]. The median minimum stenosis was 70% (range 50–70%) which was the same cutoff used in SAMMPRIS. The median technical success rate was 98% (range 93–100%). The median or mean follow-up periods were 3–13 months, and the median rate of symptomatic ISR was 3.3% (range 0–5.3%). The median rates of ipsilateral and all territory TIA or ischemic stroke were 6.2% (range 3.1–13.3%) and 9.5% (4.8–16.7%), respectively. 

One of the potential mechanisms of ISR is stimulation of intimal hyperplasia by the radial force of the stent. The Wingspan stent is an open-cell stent with a high radial opening force. In contrast, the Enterprise stent (Cordis Corporation, Miami Lakes, FL, USA) is a closed-cell, intracranial SES with reduced radial force compared to the Wingspan stent. The Enterprise stent has been used under the hypothesis that a stent with lower radial force will be less likely to result in ISR than one with high radial force. Vajda et al. reported a large series of 189 ICAD patients with 209 lesions treated with the Enterprise stent [29]. The majority of the lesions (57%) were located in the posterior circulation, the minimum degree of arterial stenosis was 50%, and 84% of patients were symptomatic. The technical success rate was 100%, and the combined rate of neurological morbidity and death was 7.7% at 30 days and 0.9% after 30 days. Major periprocedural complications were more common in the treatment of symptomatic (8.5%) compared to asymptomatic (3.1%) lesions. The rate of ISR in this study (24.7%) was not significantly different than the rate of ISR reported in the NIH Wingspan registry (25%) which comprised of data from 16 United States centers [9]. However, the proportion of the ISR lesions which were symptomatic was lower in the Enterprise study (9.3%) compared to the Wingspan registry study (15.3%). The overall rate of symptomatic ISR associated with the Enterprise stent was 2.3%.

### 3.3. Balloon-Expandable and Drug-Eluting Stents

BESs were not utilized in the SAMMPRIS trial and have not been rigorously compared to medical management or SESs in a prospective manner. Intracranial BESs are derived from coronary BESs. The majority of BESs used to treat ICAD are, in fact, coronary stents. We only identified one series in which a single, non-drug-eluting BES was used [34]. The remaining reports of BES outcomes involved multiple stent designs and types. Due to lack of demarcation in reporting outcomes between BESs and SESs in many series, the safety and efficacy of BESs alone were not always evident. Rhode et al. treated 46 patients with BESs including 35 patients with the AVE stent (Medtronic, Minneapolis, MN, USA) and 11 patients with the Neurolink stent (Guidant Corporation, Indianapolis, IN, USA). The locations of the lesions were vertebral artery in 41.3%, basilar artery in 37.0%, internal carotid artery (ICA) in 21.7%, and none in the middle cerebral artery (MCA). The minimum degree of stenosis was 50% with an average of 81%. The combined rate of stroke and death at 30 days was 23.9%, the majority of which was major stroke (13.0%).

Kurre et al. analyzed the stenting outcomes from the INTRASTENT registry which comprised of multicenter data from 18 institutions throughout Europe. BESs were used in 246 patients with 254 lesions. The locations of the lesions were ICA in 40.9%, vertebral artery in 26.4%, basilar artery in 18.1%, and MCA in 14.6%. The cumulative rate of stroke and mortality was 9.4% including nondisabling stroke (4.9%), defined as modified Rankin Scale (mRS) score less than 2, disabling stroke (3.7%), defined as mRS score at least 2, and death (0.8%). Jiang et al. treated 454 ICAD lesions located in the MCA (40.7%), basilar artery (24.7%), ICA (18.9%), and vertebral artery (15.6%) with BESs [28]. The rate of technical success was 92.9%, and the rate of periprocedural strokes was 6.0%. The mean pre- and posttreatment stenosis were 78% and 12%, respectively.

Drug-eluting stents (DES) were initially described in the cardiology literature and began to be deployed for ICAD lesions in the mid-2000s in an effort to chemically combat the high rates of ISR associated with bare metal stents [35–37]. Early studies were associated with high rates of technical failure and periprocedural complications due to the poor flexibility of the DESs in combination with the tortuous anatomy of the intracranial vasculature. However, given advances in stent design and delivery techniques since the advent of DESs for ICAD treatment, the enthusiasm for DESs appears to have returned. Currently, all DESs used for ICAD are balloon-mounted coronary stents. Vajda et al. used the Coroflex Please paclitaxel-eluting stent (B. Braun, Melsungen, Germany) to treat 95 patients with 106 ICAD lesions of at least 50% stenosis [23]. Of the treated lesions, 61% were symptomatic, 25% were associated with multiple vessel disease, and 13% were asymptomatic. All of the lesions were located proximal to the circle of Willis including in the petrous (42%), cavernous (41%), and paraclinoid (4%) ICA, basilar artery (4%), and intradural vertebral artery (10%). The technical success rate was 93.4%, and the combined rate of morbidity and mortality was 3.8% at 30 days and 0.9% beyond 30 days. The rate of ISR was an impressive 3.8%, and all cases of ISR were asymptomatic. In a smaller study of 11 patients treated with the Cypher paclitaxel-eluting stent (Cordis Corp, Johnson & Johnson, Warren, New Jersey, USA), Park et al. reported long-term outcomes over a mean follow-up period of 67 months [18]. The technical success rate was 100%, and there were no periprocedural complications, postprocedural strokes, or cases of ISR.

### 3.4. Technical Success Rates and Complications

While the rate of technical success was very high in most series, the rate of technical complications varied significantly. This is, in part, due to the variability in which events are classified as technical complications. For example, the development of intraprocedural stent thrombus which resolves with anticoagulant or antiplatelet infusion without postprocedural neurological deficit may not be reported as a complication. Another similar example is the development of intraprocedural vasospasm which resolves with calcium channel blockers without postprocedural symptoms. Tarlov et al. treated 41 patients with single ICAD lesions using either Wingspan stents or coronary BESs [26]. The rate of symptomatic groin hematoma treated with antibiotics or surgery was 14%, intraprocedural embolism requiring tissue plasminogen activator was 5%, and dissection was 23%. A study of 100 patients treated with both BESs and SESs reported 23% rate of technical complications including failure of stent placement (6%), dissection (5%), symptomatic vasospasm (5%), stent thrombosis (4%), and arterial perforation or rupture (4%) [21]. The major cause of periprocedural stroke or death (75%) in a study of 95 ICAD patients treated with the Wingspan stent was hemorrhage-related intraprocedural complications during guidewire manipulation or angioplasty [17].

### 3.5. Clinical Outcomes

While the angiographic goal of stenting is remodeling of diseased vasculature in order to revascularize inadequately perfused brain tissue, the true measure of its success is with the clinical outcome of the patient. The etiology of stroke following ICAD stenting is likely multifaceted in nature. In addition to procedural complications, poor outcomes following stenting for ICAD are largely attributable to ischemic stroke resulting in neurological morbidity and mortality. The causes of ischemic stroke include ISR, progression of ICAD in treated and untreated vessels, occlusion of perforator branches, and distal thromboembolism. Hemorrhagic strokes may occur, outside the setting of intraprocedural vessel perforation, secondary to reperfusion, hemorrhagic conversion of a prior ischemic stroke, or at distant sites without a clear etiology. 

Detailed analysis of the European INTRASTENT registry demonstrated the cause of ischemic and hemorrhagic strokes resulting in clinical disability [31]. Stroke secondary to perforator branch occlusion occurred in 10 patients (2.5%) and was disabling in seven. Thromboembolic infarcts occurred in six patients (1.5%) and was disabling in three patients. Stent thrombosis occurred in four patients (1.0%) but was only disabling in one. Reperfusion hemorrhage occurred in four patients (1.0%) and resulted in disability in two patients and mortality in two patients. Hemorrhagic conversion of a prior ischemic stroke occurred in one patient (0.3%) and was disabling. Hemorrhage of unknown etiology occurred in three patients (0.8%) all of which resulted in mortality. Due to their relatively infrequent occurrences, determining predictors of these events is difficult and may never be achieved given the tenuous future of stenting for ICAD.

### 3.6. In-Stent Restenosis following Stenting for Intracranial Atherosclerosis

ISR occurs with significant frequency following stenting for ICAD [8]. However, most cases of ISR are asymptomatic. Symptomatic ISR may be managed conservatively with continued antiplatelet therapy and close angiographic surveillance, or it may be treated with further intervention, such as angioplasty or repeat stenting, in select cases of progressive neurological decline. Rigorous angiographic followup is necessary for the detection of ISR. It remains unknown whether technical attempts to reduce ISR by utilizing SESs with lower radial force or DESs have resulted in significant decreases in its occurrence [18, 23, 29]. 

Jin et al. reviewed the follow-up angiography of 226 patients with 233 stented lesions and found a significantly higher rate of TIA or stroke in the cohort of patients with ISR compared to the cohort of patients without ISR (21.1% versus 8.5%, *P* = 0.005) [33]. Additionally the patients with ISR developed TIA or stroke significantly earlier than the patients without ISR (9.9 versus 26.6 months, *P* = 0.01). Multivariate analysis identified ISR to be an independent predictor of TIA or stroke following stenting for ICAD (*P* = 0.017). The rate of postprocedural ischemic events was not found to be significantly different between symptomatic and asymptomatic ISR patients (*P* = 0.96). Zhang et al. determined that a ratio of reference artery to stent diameter of less than 0.78 and length of vascular lesion less than 5.39 mm were significantly associated with increased incidence of ISR (*P* = 0.013 for both variables) [25]. Identifying methods to minimize the occurrence of ISR has the potential to substantially improve ICAD stenting outcomes.

## 4. Discussion

### 4.1. The SAMMRPIS Trial and Its Criticisms

SAMMPRIS was a multicenter, prospective, randomized clinical trial which enrolled patients with recent (i.e., within 30 days) transient ischemic attack (TIA) or nondisabling stroke which could be attributed to 70–99% stenosis of a major intracranial artery [10]. Patients were randomized to aggressive medical management with or without intervention. Both cohorts received medical therapy which consisted of dual antiplatelet therapy (i.e., aspirin 325 mg daily and clopidogrel 75 mg daily) and treatment of risk factors including hypertension (goal systolic blood pressure less than 140 mm Hg) and hypercholesterolemia (goal low-density lipoprotein cholesterol levels less than 70 mg/dL). Endovascular intervention consisted of initial angioplasty of the diseased arterial segment with the Gateway PTA balloon catheter followed by subsequent deployment of a Wingspan stent. The study's primary end point was stroke or death within 30 days of enrollment or intervention or ischemic stroke in the territory of the diseased artery between day 31 and the end of the follow-up duration.

A total of 451 patients were randomized to medical therapy only (*N* = 227) or intervention (*N* = 224). Of the patients randomized to intervention, 16 (7.1%) did not undergo stent placement, and of the patients randomized to medical therapy, nine (4.0%) underwent intervention for subsequent TIA. The primary end point was observed within 30 days in 33 patients in the intervention cohort (14.7%) compared to 13 patients in the medical cohort (5.7%), demonstrating a significantly better outcome in the medical cohort (*P* = 0.002). A higher proportion of 30-day strokes in the intervention cohort were intracerebral hemorrhages (30.3% versus 0%, *P* = 0.04). At one-year followup, the occurrence rate of the primary end point remained higher in the intervention cohort compared to the medical cohort (20.0% versus 12.2%, *P* = 0.009). In SAMMPRIS, the periprocedural stroke rate following angioplasty and stenting was higher than expected and the stroke rate from medical management was lower than expected.

There have been concerns raised regarding the operator experience of the SAMMPRIS neurointerventionalists [38]. Yu et al. noted a decrease in the rates of technical failure and intraprocedural complications during treatment of ICAD with the Wingspan stent, including guidewire- and angioplasty-related hemorrhages, as operator experience at their institution increased although the difference was not statistically significant [17]. Derdeyn et al. examined the effect of operator and site experience during the SAMMPRIS trial on stenting outcomes [39]. All interventions in the SAMMPRIS trial were performed by 63 neurointerventionalists at 48 sites, and the median number of Wingspan stent procedures submitted for credentialing by each neurointerventionalist was 10 (range 3–20). The median number of procedures performed by each neurointerventionalist was 3 (range 1–13). There were 34 periprocedural complications comprised of 19 ischemic strokes, 11 symptomatic hemorrhagic strokes, two ischemic events with temporary symptoms, and two asymptomatic hemorrhagic strokes. There was no significant association between periprocedural complications and neurointerventionalist or site features. In fact, the periprocedural complication rate for neurointerventionalists credentialed with less than 10 Wingspan procedures was 9.9% compared to 19.0% for those credentialed with at least 10 Wingspan procedures although this difference was not statistically significant (*P* = 0.11).

Another major criticism of SAMMPRIS has been the significant proportion of patients who had not failed medical therapy prior to endovascular intervention [10]. The Wingspan stent was approved by the FDA and generally intended for use in patients who had failed medical management [7, 8]. However, 35.3% of the stenting cohort in SAMMPRIS had not received antithrombotic therapy at the time of the qualifying clinical event. In current clinical practice, patients routinely receive at least one antiplatelet agent, most commonly aspirin 325 mg daily, for secondary stroke prevention following the occurrence of a TIA or ischemic stroke. Therefore, in a practical setting, it is unlikely that a patient who presents with symptomatic ICAD would proceed to endovascular treatment without an initial trial period of antithrombotic therapy [40].

Despite its criticism, the ramifications of SAMMPRIS on the current management of ICAD have been significant in the two years since its publications. Zaidat et al. conducted a poll of 217 attendees at the 2012 ICAD symposium during the 2012 International Stroke Conference to assess the effect of SAMMPRIS on clinical practice and perspectives regarding the management of ICAD [41]. Neurologists, neurointerventionalists, and neurosurgeons comprised 71% of the audience with a predominance of neurologists (57%). The average response rate for each question was 82%. Audience responses of note were as follows: 58% responded that SAMMPRIS had diminished their enthusiasm for stenting of ICAD, 84% agreed with the medical management undertaken in SAMMPRIS, only 28% still recommended treatment of an ICAD patient who failed aggressive medical therapy with angioplasty and stenting, and 86% believed further clinical trials were needed for ICAD management.

### 4.2. Effect of Stent Type on ICAD Treatment Outcomes

BESs are typically less malleable than SESs and therefore can be more difficult to navigate through tortuous vascular anatomy. The deployment of an SES is preempted by submaximal dilatation with balloon angioplasty. The target lesion must be traversed twice by a microcatheter for an SES: the first time for balloon predilatation and the second time for stent deployment. In contrast, both of these steps are achieved simultaneously for a BES which only requires traversing the lesion with a microcatheter once. While this difference in stent deployment technique theoretically favors BESs over SESs in regard to periprocedural safety, a significant discrepancy in intraprocedural or periprocedural complications has not been noted between the two stent designs. In a study of 41 patients, Tarlov et al. did not find a difference in postprocedural stroke rate between patients treated with the Wingspan stent (*N* = 19) and those treated with various balloon-mounted coronary stents (*N* = 22, *P* = 0.819) [26].

In a large multicenter, retrospective analysis comprised of 637 patients with 670 ICAD lesions treated with multiple BES compared to the self-expanding Wingspan stent, Jiang et al. found that patients treated with BES had significantly lower degrees of posttreatment stenosis (*P* = 0.001) and were less likely to develop ISR (*P* = 0.02) at three- and six-month followup [28]. On the contrary, BES had a higher rate of technical failure than Wingspan stents (7.1% versus 1.4%, *P* < 0.001). The same study identified Mori type A lesion and presence of higher degrees of postprocedural stenosis to be predictors of ISR development at followup (*P* < 0.001 and *P* = 0.006, resp.). There was no difference between BES and Wingspan stent with regard to the rate of periprocedural stroke (*P* = 0.46). In 100 consecutive patients treated with BES (*N* = 46) or SES (*N* = 54), Rohde et al. found a lower rate of vascular complications with SES compared to BES (11.1% versus 36.9%, *P* = 0.002) but no statistically significant difference in rates of technical success (96.3% for SES versus 89.1% for BES, *P* = 0.31) or combined stroke and mortality rate at 30 days (25.9% for SES versus 23.9% for BES) [21]. Kurre et al. also did not find a difference in postprocedural adverse events, including stroke and death, when comparing BES to SES in 397 patients with 409 lesions retrospectively collected from a multicenter registry [31]. 

### 4.3. Defining the Current and Future Roles of Stenting for the Treatment of Intracranial Atherosclerosis

Despite the waning enthusiasm for the treatment of ICAD with stents, a role for stenting ICAD lesions remains. However, the burden lies with the neurointerventional and cerebrovascular communities to define the proper patient population for current interventional therapies and, more importantly, to prove in well-designed, prospective, randomized trials, such as SAMMPRIS, that the safety and efficacy of stenting has not been underestimated. Several factors should also be taken into consideration when planning future studies, including the role of angioplasty without stenting, the outcomes of SESs versus BESs, the role of DESs, and the focused analysis of subgroups based on the location and morphology of the ICAD lesion.

While BESs are generally associated with angiographically superior and more durable results than SESs, including lower rates of ISR, they remain less versatile due to their stiffer nature. Furthermore, current data comparing BESs to SESs is limited by its retrospective nature. Despite both theoretical and reported advantages and disadvantages between BESs and SESs, there has yet to be a well-designed trial which compares one stent design to the other. As advances in endovascular technology continue to be made at a rapid pace, angioplasty without stenting, which was largely abandoned after popularization of the Wingspan stent in the late 2000s, may prove to be a safer alternative to stenting while providing equivalent efficacy [42, 43]. However, this has yet to be proven in a rigorous fashion, and this treatment option should be considered when designing future ICAD trials.

It is understandably difficult to organize large scale prospective studies. In order to maximize the utility of retrospective studies, it is important that the reporting guidelines for all ICAD stenting studies become standardized. The most widely used angiographic classification of ICAD lesions was described by Mori et al. which divided them into three types [44, 45]. Mori type A lesions are short (5 mm or less in length), concentric or moderately eccentric, and nonocclusive; type B lesions are tubular (5–10 mm in length), extremely eccentric or occlusive, and less than three months old; type C lesions are diffuse (greater than 10 mm in length), angulated greater than 90 degrees, extremely tortuous in their proximal segments or completely occluded, and at least three months old. The risk of ipsilateral ischemic stroke was 8%, 12%, and 56% in patients with Mori types A, B, and C lesions, respectively [44]. The MCA is the most commonly affected location for ICAD, accounting for 32.4% of patients in the WASID study and 43.7% of patients in the SAMMRPIS study including 41.1% of patients in the stenting cohort [4, 10]. Due to its distal location relative to the ICA and vertebrobasilar system, stenting an MCA lesion is also relatively more difficult and prone to intraprocedural complication. Arteries with relatively dense and critical perforator branch arteries, such as the lenticulostriate branches of the proximal MCA M1 segment and the pontine perforators of the midbasilar artery are prone to thrombotic occlusion after stenting. Future studies have the potential to identify specific therapies which may be associated with more favorable outcomes for lesions of a specific artery or Mori classification. 

Ultimately, further basic and translational science efforts are necessary to better bridge fundamental knowledge gaps in the pathogenesis and progression of ICAD lesion as well as their rupture which leads to end-stage events such as thromboembolism and stroke. Identification of unstable atherosclerotic plaques through novel neuroimaging techniques, such as intravascular ultrasound, is a potentially powerful diagnostic tool which has yet to be clinically validated [46, 47]. Animal models of unstable atherosclerotic plaques may provide insight into the molecular underpinnings of atherosclerosis and mechanisms by which its progression may be halted or even reversed [48].

### 4.4. Study Limitations

This review is limited by the retrospective nature of the ICAD stenting series it encompasses. The variability in the outcomes and followup reported in each of the series makes comparisons across different patient, lesion, and stent characteristics difficult and unreliable. This study's major limitation is the timing of many of the series. Despite our intention to report ICAD stenting outcomes after the SAMMPRIS trial by including only those studies published after SAMMPRIS, there was likely a six- to 12-month period of publication overlap following SAMMPRIS in which new studies describing ICAD stenting outcomes did not account for the results from SAMMPRIS. This is due to the time difference in various journals' manuscript review and production periods and in authors' time to manuscript revision following review. Additionally, many of the stenting studies published after SAMMPRIS included patients treated prior to 2011 and therefore are not representative of the treatment biases which arose following the dissemination of the SAMMPRIS trial results. Presently, more time is necessary for the effect of SAMMPRIS on ICAD management strategies to be fully recognized and accounted for in the literature.

## 5. Conclusions

Patients with symptomatic, severe ICAD represent a very high-risk cohort who harbor relatively high prospective risks of recurrent ischemic events. Stenting of ICAD lesions was initially postulated to positively impact the natural history of ICAD by reducing the risk of neurological morbidity secondary to stroke. Surprisingly, the SAMMPRIS trial demonstrated an overestimation of the benefits of angioplasty and stenting with the Wingspan stent system and an underestimation of the efficacy of aggressive medical management. However, stenting for ICAD continues to be performed albeit with significantly more reserve since the release of SAMMPRIS. While results with newer SES systems, BESs, and DESs are promising with regard to relatively low rates of ischemic events and symptomatic ISR, future ICAD trials are needed to rigorously compare across various stent designs and to compare current stenting practices to angioplasty alone and to medical management. Despite maximal medical therapy, patients with significant ICAD may continue to experience TIAs and strokes associated with disease progression. Until novel medical therapies are shown to improve the poor outcomes associated with symptomatic ICAD, endovascular stenting will continue to play a role in the treatment of a carefully selected subset of ICAD patients.

## Figures and Tables

**Table 1:** Summary of endovascular stenting series for the treatment of intracranial atherosclerosis published after SAMMPRIS.

Series	Year	Type of stent	Number of patients (lesions)	Minimum arterial stenosis	Technical success rate	Mean/median clinical follow-up duration (months)	Rate of postprocedural ischemia (ipsilateral)	Rate of postprocedural ischemia (all)	Rate of symptomatic in-stent restenosis
Gandini et al. [20]	2013	Wingspan	21 (42)	70%	100%	20	4.8%	4.8%	0
Jin et al. [33]	2013	Multiple^1^	226 (233)	NR	NR	39	11.6%	11.6%	5.2%
Park et al. [18]	2013	Cypher	11 (11)	50%	100%	67	0	0	0
Rohde et al. [21]	2013	Multiple^2^	100 (100)	50%	94.0%	1	NR	25%	NR
Yu et al. [17]	2013	Wingspan	95 (95)	70%	93.7%	41	3.1% in 30 days, 0% from 30 days to 1 year	9.5% in 1 year	NR
Zhang et al. [19]	2013	Wingspan	61 (61)	70%	98.4%	24	10.0%	11.7%	3.3%
Jiang et al. [28]	2012	Multiple^3^	637 (670)	50%	94.6%	NR	5.8% at 30 days, 13.7% overall	5.8% at 30 days, 13.7% overall	11.1%
Kim et al. [22]	2012	Multiple^4^	77 (85)	50%	87.1%	29	7.7%	9.2%	3.1%
Kurre et al. [31]	2012	Multiple^5^	397 (409)	50%	98.0%		NR	11.6%	NR
Li et al. [30]	2012	Wingspan	30 (31)	70%	100%	18	13.3%	16.7%	5.3%
Mohammadian et al. [24]	2012	Multiple^6^	34 (34)	70%	97.0%	15	2.9%	5.8%	0
Tarlov et al. [26]	2012	Multiple^7^	41 (41)	50%	100%	14	NR	20.0%	7.5%
Vajda et al. [29]	2012	Enterprise	189 (209)	50%	99.5%	7	2.3%	7.7%	2.3%
Vajda et al. [23]	2012	Coroflex Please	95 (106)	50%	93.4%	16	1.9%	3.8% in 30 days, 0.9% after 30 days	0
Yu et al. [27]	2012	Wingspan	57 (57)	50%	93.0%	NR	NR	8.8%	NR
Zhang et al. [25]	2012	Wingspan	53 (53)	50%	98.1%	18	6.0%	10.0%	4.0%
Costalat et al. [32]	2011	Wingspan	60 (63)	50%	95.2%	13	6.3%	6.3%	2.0%
Park et al. [34]	2011	FlexMaster	12 (14)	70%	100%	9	0	0	0

NR: not reported.

^1^Multiple stents were used in this study including Wingspan (Boston Scientific), Apollo (MicroPort Medical), and coronary balloon-expandable stents.

^2^Multiple stents were used in this study including AVE (Medtronic), Neurolink (Guidant), Neuroform (Boston Scientific), and Wingspan (Boston Scientific) stents.

^3^Multiple stents were used in this study including Vision (Abbott), Mini-Vision (Abbott), Penta (Abbott), Taxus Express (Boston Scientific), Cypher (Cordis Corp), S70 (Medtronic), Driver (Medtronic), Apollo (MicroPort Medical), BiodivYsio (Biocompatibles), and Wingpan (Boston Scientific) stents.

^4^Multiple stents were used in this study including Endeavor (Medtronic), Vision (Abbott), FlexMaster (Abbott), Arthos pico (AMG International), Neuroform (Boston Scientific), Tsunami (Terumo), and Driver (Medtronic) stents.

^5^Multiple stents were used including Wingspan (Boston Scientific), LEO (BALT Extrusion), Neuroform (Boston Scientific), Enterprise (Cordis Corp), Xpert (Abbott), Pharos (Micrus), BOA (BALT Extrusion), Apollo (MicroPort Medical), Arthos (AMG), AVE S660 and S670 (Medtronic) BX Sonic and Velocity (Cordis Corp), Cerebrence (Medtronic), Coroflex blue (Braun), Driver (Medtronic), Endeavour (Medtronic), FlexMaster (Abbott), INX (Medtronic), Lekton Motion (Biotronik), Multi-Link (Abbott), S7 (Medtronic), Taxus Express and Liberte (Boston Scientific), Tecnic carbostent (Sorin Biomedica), and Tsunami Gold (Terumo) stents.

^6^Multiple stents were used including balloon-mounted, coronary bare metal stent for internal carotid artery stenosis, and self-expanding stents for middle cerebral artery stenosis.

^7^Multiple stents were used including Wingspan (Boston Scientific), AVE (Medtronic), ACS, Voyager (Abbott), Vision (Abbott), Palmaz-Schatz (Cordis Corp), Multilink (Abbott), and other Guidant and Medtronic stents.

**Table 2:** Stent types and manufacturers used in endovascular stenting series published after SAMMPRIS.

Manufacturer	Location	Stent name	Stent design*	Stent type	Eluting drug
Abbott Vascular	Abbott Park, IL, USA	FlexMaster	BES	Coronary	None
		Mini Vision	BES	Coronary	None
		Multi-Link	BES	Coronary	None
		Penta	BES	Coronary	None
		Vision	BES	Coronary	None
		Voyager	BES	Coronary	None
		Xpert	SES	Biliary	None
AMG International GmbH	Raesfeld-Erle, Germany	Arthos Pico	BES	Coronary	None
BALT Extrusion	Montmorency, France	Boa	BES	Intracranial	None
		LEO	SES	Intracranial	None
Biocompatibles	San Jose, CA, USA	BiodivYsio	BES	Coronary	Dexamethasone
Biotronik	Bulach, Switzerland	Lekton Motion	BES	Coronary	None
Boston Scientific	Natick, MA, USA	Neuroform	SES	Intracranial	None
		Taxus Express	BES	Coronary	Paclitaxel
		Taxus Liberte	BES	Coronary	Paclitaxel
		Wingspan	SES	Intracranial	None
B. Braun Medical	Melsungen, Germany	Coroflex Blue	BES	Coronary	None
		Coroflex Please	BES	Coronary	Paclitaxel
Cordis Corporation	Miami Lakes, FL, USA	BX Sonic	BES	Coronary	None
		BX Velocity	BES	Coronary	None
		Cypher	BES	Coronary	Sirolimus
		Enterprise	SES	Intracranial	None
		Palmaz-Schatz	BES	Coronary	None
Guidant Corporation	Indianapolis, IN, USA	Neurolink	BES	Intracranial	None
Medtronic	Minneapolis, MN, USA	AVE	BES	Biliary	None
		AVE S660	BES	Coronary	None
		AVE S670	BES	Coronary	None
		Cerebrence	BES	Coronary	None
		Driver	BES	Coronary	None
		Endeavor	BES	Coronary	Zotarolimus
		INX	BES	Intracranial	None
		S7	BES	Coronary	None
		S70	BES	Coronary	None
MicroPort Medical	Shanghai, China	Apollo	BES	Intraranial	None
Micrus Endovascular Corporation	Sunnyvale, CA, USA	Pharos	BES	Intracranial	None
Sorin Biomedica	Saluggia, Italy	Tecnic Carbostent	BES	Coronary	None
Terumo Corporation	Tokyo, Japan	Tsunami	BES	Coronary	None
		Tsunami Gold	BES	Coronary	None

*BES: balloon-expandable stent; SES: self-expanding stent.
